# Macrophage states and polarization during *Mycobacterium tuberculosis* infection: mechanisms, granuloma adaptation, and host-directed therapeutic opportunities

**DOI:** 10.3389/fcimb.2026.1913094

**Published:** 2026-09-04

**Authors:** Minglong Xu, Zhangli Peng

**Affiliations:** Department of General Practice, Affiliated Hospital of Zunyi Medical University, Zunyi, Guizhou, China

**Keywords:** epigenetic reprogramming, granuloma, host-directed therapy, macrophage polarization, *Mycobacterium tuberculosis*, nanocarrier delivery

## Abstract

Tuberculosis is a chronic infectious disease caused by *Mycobacterium tuberculosis* (Mtb). As primary host cells targeted by Mtb, macrophages play central roles in both innate and adaptive immunity. Accumulating evidence indicates that macrophage polarization is a key determinant of tuberculosis pathogenesis. In response to diverse microenvironmental cues, macrophages adopt functionally distinct polarization states, ranging from pro-inflammatory, microbicidal programs (M1-like) to anti-inflammatory, tissue-reparative programs (M2-like). These states differentially shape tuberculosis progression. Defining the remodeling of these context-dependent macrophage states is therefore critical for understanding Mtb infection, granuloma formation, and disease outcomes, as well as for guiding the development of next-generation vaccines, immunotherapies, and host-directed interventions. This Review synthesizes the molecular mechanisms underlying macrophage polarization during Mtb infection, integrates the bidirectional regulatory networks between pathogen-derived and host-derived factors, and highlights their roles in immune evasion, granuloma biology, and emerging therapeutic strategies in tuberculosis.

## 1 Introduction

Tuberculosis is a chronic infectious disease caused by *Mycobacterium tuberculosis* (Mtb). According to the World Health Organization Global Tuberculosis Report 2025, an estimated 10.7 million incident cases were reported worldwide in 2024, corresponding to an incidence of 131 per 100,000 population (World Health Organization, 2025). Although both incidence and absolute case numbers declined compared with 2023, the global burden of tuberculosis remains substantial. China continues to rank among high-burden countries and accounts for the fourth highest number of incident cases globally. Despite a reduction in tuberculosis-related deaths to 1.23 million in 2024, tuberculosis remains the leading cause of death from a single infectious agent. Collectively, these data underscore the persistent global health challenge posed by tuberculosis and highlight the need for improved prevention and control strategies.

As the first line of defense against Mtb infection, macrophages exhibit remarkable plasticity and differentiate into distinct functional phenotypes in response to microenvironmental cues (Khan et al., 2019; Ahmad et al., 2022), a process known as macrophage polarization. During infection, macrophages not only mediate pathogen uptake and intracellular killing, but also shape downstream adaptive immunity through antigen presentation and cytokine production (Chang et al., 2005).

In the steady-state lung, macrophages comprise alveolar macrophages (AMs) and interstitial macrophages (IMs) (Bain and MacDonald, 2022). AMs arise predominantly from fetal monocytes, undergo granulocyte–macrophage colony-stimulating factor (GM-CSF)– and peroxisome proliferator-activated receptor-γ (PPAR-γ)-dependent differentiation, and are maintained largely through local self-renewal under homeostatic conditions (Guilliams et al., 2013; Schneider et al., 2014). Their transcriptional and epigenetic identity is continuously shaped by the alveolar microenvironment, enabling efficient surfactant catabolism, clearance of cellular debris, and restraint of excessive inflammatory responses. In mice, AMs are commonly characterized as CD11c^high^ Siglec-F^high^ CD11b^low^ cells, whereas IMs generally express CD64, MerTK, and CD11b but lack or express relatively low levels of Siglec-F (Aegerter et al., 2022; Bain and MacDonald, 2022). However, these phenotypic markers and subset definitions vary across species, anatomical locations, and disease states.

In contrast to the relatively developmentally stable AM compartment, IMs are ontogenetically and functionally heterogeneous. They include embryonically seeded, self-renewing populations as well as monocyte-derived macrophages that are recruited or replenished to varying degrees during inflammation. Distinct IM subsets occupy specialized perivascular, peribronchial, nerve-associated and alveolar-interstitial niches, where they exhibit different antigen-presenting, phagocytic, inflammatory and immunoregulatory properties (Schyns et al., 2019). During Mtb infection, however, macrophage ontogeny and anatomical localization do not map directly onto fixed M1 or M2 phenotypes. AMs are among the earliest pulmonary cells infected after inhalation and may provide a relatively permissive niche for initial bacillary replication and dissemination (Cohen et al., 2018). As infection progresses, recruited monocyte-derived and interstitial macrophage populations become increasingly prominent and may display stronger antigen-presenting, inflammatory or antimicrobial programs. Nevertheless, macrophages within both compartments can adopt mixed and transitional states shaped by developmental origin, local cytokine milieu, bacterial burden, T-cell-derived signals, hypoxia, nutrient availability and spatial position within the developing granuloma (Huang et al., 2018).

Foamy macrophages fit within this continuum as a lipid-laden metabolic and functional state rather than as a separate macrophage lineage or a synonym for the M2 phenotype. Lipid-droplet accumulation can reshape macrophage inflammatory and antimicrobial functions while creating a nutrient-rich intracellular environment that favors Mtb adaptation and persistence (Bedard et al., 2023; Chen et al., 2024). Therefore, within the simplified M1/M2 framework, classically activated M1-like macrophages are useful descriptors of pro-inflammatory and antimicrobial programs, whereas alternatively activated M2-like macrophages broadly describe programs associated with inflammation resolution, immune regulation and tissue repair (Liu et al., 2017). *In vivo*, these labels should be interpreted as functional reference states rather than discrete cellular identities, because macrophages frequently exhibit overlapping or intermediate features and undergo dynamic transitions in response to tissue and lesion-specific cues. These context-dependent macrophage states contribute to the balance among bacterial control, chronic persistence, tissue repair and immunopathology, but no single state alone determines disease outcome.

However, as a highly adapted intracellular pathogen, Mtb can subvert macrophage functional states to promote immune evasion and establish chronic infection (Zhai et al., 2019). Rather than inducing a uniform transition toward a single polarization phenotype, Mtb may simultaneously enhance immunoregulatory or tissue-remodeling programs, reprogram host lipid metabolism to facilitate intracellular persistence, and suppress antimicrobial effector mechanisms, including the generation of reactive oxygen and nitrogen species (Genoula et al., 2018). These responses are further regulated by host epigenetic and post-transcriptional programs, including microRNA networks and histone modifications, and by the spatially heterogeneous microenvironment of the granuloma (Thomas et al., 2024). However, the molecular mechanisms through which Mtb coordinates these context-dependent macrophage state transitions, and the extent to which specific macrophage states promote bacterial control, persistence, or tissue injury, remain incompletely understood. This Review therefore uses M1-like and M2-like terminology as an organizing framework while emphasizing macrophage plasticity, intermediate activation states and lesion-specific phenotypes during Mtb infection. We summarize the pathogen- and host-derived mechanisms that regulate macrophage states, examine their adaptation within the granuloma microenvironment, and discuss their implications for immune evasion and host-directed therapeutic strategies in tuberculosis.

## 2 Overview of macrophage polarization

Macrophages are highly plastic innate immune cells that play central roles in antimicrobial defense, inflammatory regulation, and tissue homeostasis (Chen et al., 2023). In response to environmental cues, macrophages undergo coordinated transcriptional, metabolic, and functional reprogramming, a process commonly referred to as macrophage polarization (Murray et al., 2014). *In vitro*, the classical M1/M2 framework remains a useful conceptual model for describing polarized macrophage functions, especially in controlled experimental settings. Within this framework, M1-like programs, typically induced by IFN-γ and microbial stimuli, are associated with inflammatory cytokine production, reactive oxygen species generation, and nitric oxide-dependent antimicrobial activity (Mantovani et al., 2004; Murray et al., 2014). By contrast, M2-like programs, often induced by IL-4 and IL-13, are associated with tissue remodeling, anti-inflammatory and reparative functions, and increased expression of markers such as CD206 and ARG-1.

However, macrophage polarization is now recognized as a dynamic and heterogeneous continuum rather than a rigid binary process. *In vivo* macrophage states are shaped by local cytokine signals, metabolic stress, tissue-specific cues, pathogen burden, and spatial niche. Therefore, M1-like and M2-like labels should be interpreted as simplified descriptors of dominant functional programs rather than as fixed cellular identities. Systems-level studies, including single-cell RNA sequencing and spatial transcriptomics, further show that infected lung macrophages comprise multiple tissue-resident, recruited, foamy, interferon-responsive, lipid-remodeled, and lesion-adapted states rather than two mutually exclusive subsets (Pisu et al., 2021; Qiu et al., 2024; Samanta et al., 2024). Similarly, intermediate or hybrid activation states have been described in complex pathological microenvironments, highlighting the adaptability of macrophages beyond conventional M1/M2 categories (Ge et al., 2021; Parisi et al., 2018; Samanta et al., 2024). Although M2-like programs have historically been subdivided into functional subsets according to inducing stimuli and effector profiles, these classifications should also be viewed as simplified experimental models rather than discrete *in vivo* populations.

Recent single-cell and spatial studies provide concrete examples of this continuum in tuberculosis. In Mtb-infected mouse lungs, reporter-based single-cell RNA sequencing resolved infected alveolar macrophages and multiple interstitial macrophage populations, linking macrophage lineage and activation state to bacterial stress, drug-tolerance-associated phenotypes, and intracellular bacterial fitness (Pisu et al., 2021). Spatial transcriptomic profiling of human lung and omental granulomas further showed that macrophage-enriched regions are embedded within distinct lesion microenvironments characterized by variable immune-cell composition, fibroblast and B-cell infiltration, extracellular-matrix remodeling, and necrotic or parenchymal tissue contexts (Qiu et al., 2024). These findings support a lesion-specific view in which M1-like and M2-like signatures are best understood as reference programs superimposed on macrophage ontogeny, metabolic adaptation, and spatial niche, rather than as discrete endpoints.

**Figure 1:**
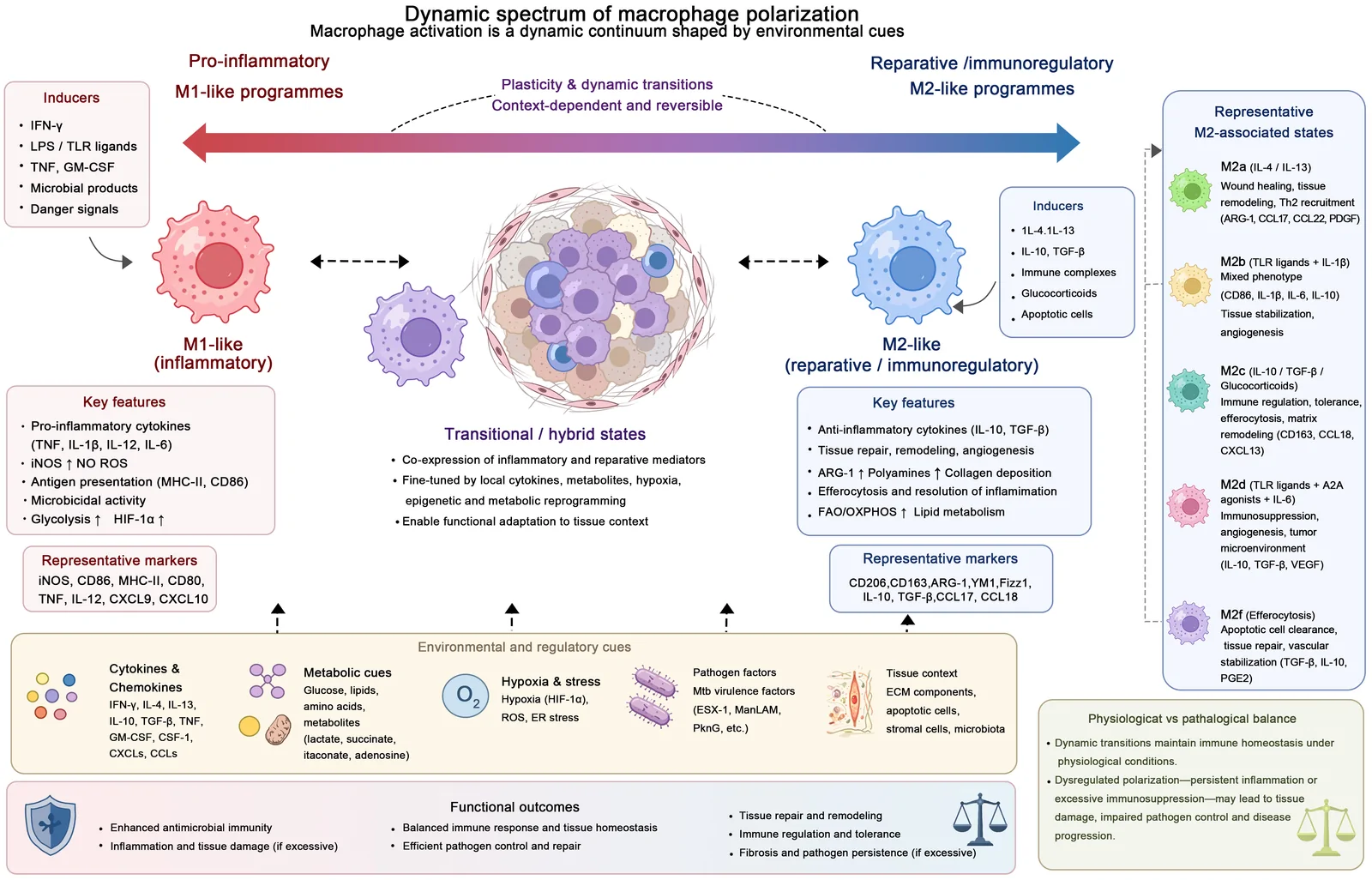
Dynamic spectrum of macrophage activation. M1-like and M2-like programs are presented as heuristic reference poles representing predominantly inflammatory–antimicrobial and reparative–immunoregulatory functions, respectively, rather than as mutually exclusive lineages or fixed *in vivo* subsets. Macrophages may adopt overlapping, transitional, and reversible states in response to cytokines, metabolic cues, hypoxia, microbial factors, and tissue-specific signals. The M2a–M2f categories summarize historically defined stimulus-associated programs and should not be interpreted as discrete macrophage populations. Marker expression is illustrative and may vary across species, tissues, disease stages, and experimental models. During *Mycobacterium tuberculosis* infection, these functional programs are superimposed on macrophage ontogeny, anatomical niche, and metabolic adaptation, thereby influencing antimicrobial defense, tissue injury, repair, and bacterial persistence.

In tuberculosis, this plasticity is particularly important because Mtb actively manipulates macrophage signaling, metabolism, and epigenetic programs to reshape host immune responses. Dynamic transitions among inflammatory-antimicrobial, immunoregulatory-reparative, foamy, and spatially adapted macrophage states are therefore increasingly recognized as central determinants of bacterial persistence, granuloma evolution, and disease outcome. This continuum is illustrated in **Figure 1**.

## 3 *Mycobacterium tuberculosis*-derived regulators of macrophage polarization

**Figure 2:**
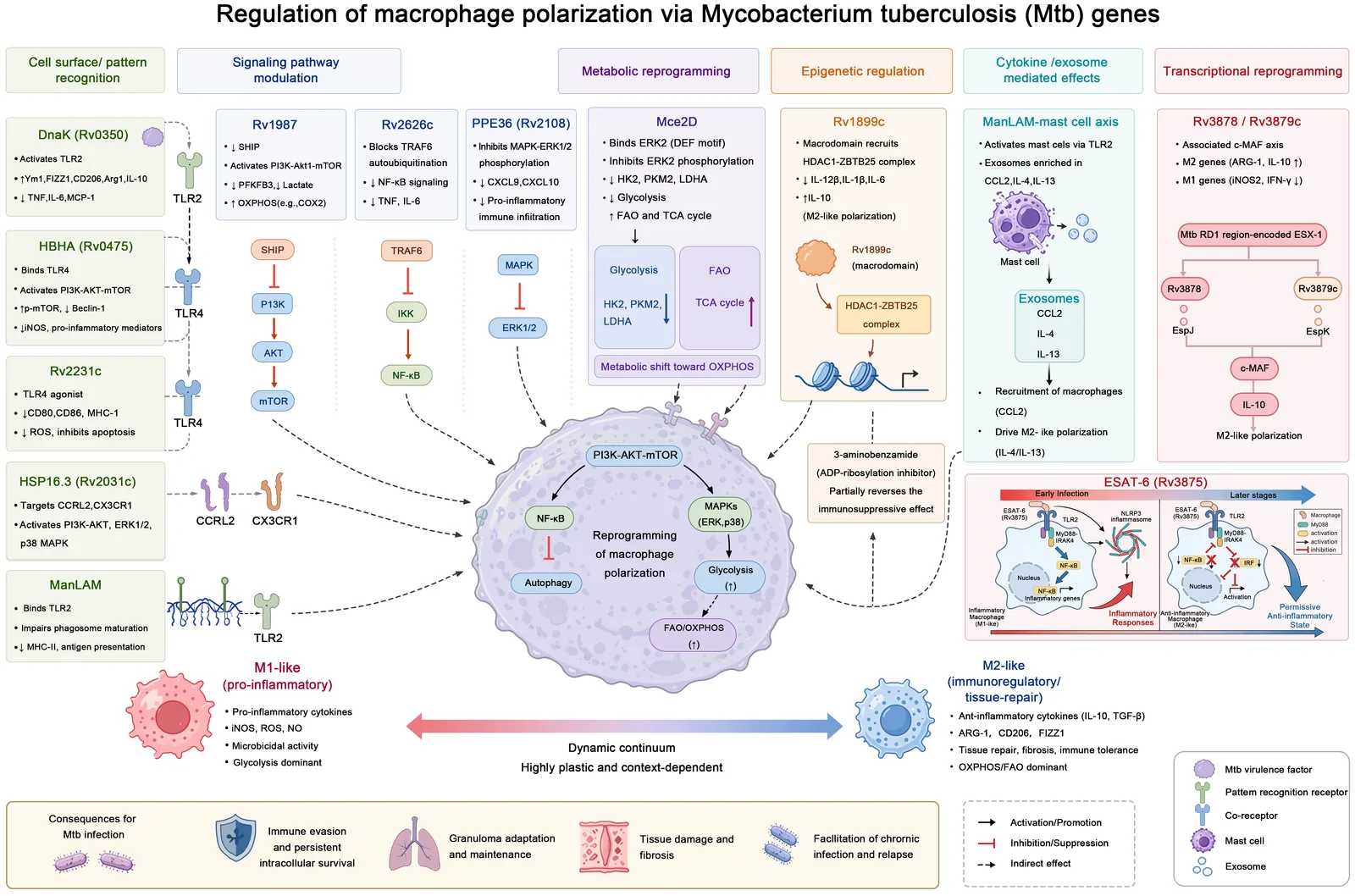
Regulation of macrophage polarization by *Mycobacterium tuberculosis*-related genes and virulence factors. The schematic shows how Mtb-derived components shape macrophage functional-state trajectories, with M1-like and M2-like features used as reference programs to summarize dominant functional features, rather than fixed endpoints. Representative bacterial effectors and cell-wall components, including Rv1987, Rv2231c, HBHA (Rv0475), DnaK (Rv0350), ManLAM, PPE36 (Rv2108), ESAT-6 (Rv3875), HSP16.3 (Rv2031c), Rv2626c, EspJ (Rv3878), EspK (Rv3879c) and Rv1899c, engage host receptors or intracellular regulators to modulate PI3K–AKT–mTOR, SHIP, TLR2/TLR4, ERK, p38 MAPK, c-MAF, NLRP3/ASC, reactive oxygen species and autophagy-related signaling. These signals shape macrophage functional programs along inflammatory, antimicrobial, immunoregulatory and tissue-remodeling axes, including M2-associated markers such as ARG-1, IL-10, Ym1 and FIZZ1 and M1-associated markers such as iNOS, IL-6, IL-12, TNF-α and IL-1β. The figure highlights multilayered manipulation of host macrophage programs along inflammatory, antimicrobial, immunoregulatory and tissue-remodeling axes.

*Mycobacterium tuberculosis* (Mtb) has evolved sophisticated immune-evasion strategies that extensively reprogram macrophage functional states. Rather than acting through a single pathway, Mtb-derived virulence factors coordinately target inflammatory signaling, cellular metabolism, autophagy and epigenetic regulation to suppress antimicrobial programs while promoting immunoregulatory and tissue-remodeling phenotypes. These interconnected processes collectively facilitate immune evasion, intracellular persistence and chronic infection, as illustrated in **Figure 2**.

Multiple Mtb virulence factors converge on host inflammatory signaling pathways to suppress antimicrobial macrophage activation and establish permissive intracellular niches. The chaperone DnaK (Rv0350), one of the best-characterized immunomodulatory proteins produced by Mtb, activates TLR2-associated signaling and promotes acquisition of M2-like immunoregulatory features, accompanied by increased expression of ARG-1, IL-10, Ym1 and FIZZ1 (Lopes et al., 2014). Similarly, HBHA (Rv0475) activates PI3K–AKT–mTOR signaling and suppresses autophagy-associated antimicrobial programs, thereby shifting macrophages away from microbicidal phenotypes and toward immune-tolerant states that favor persistent intracellular infection (Zheng et al., 2024). Rv2231c also contributes to immune suppression by reducing reactive oxygen species production and inhibiting macrophage apoptosis, whereas HSP16.3 (Rv2031c) promotes integrated signaling responses associated with M2-like, immune-tolerant reprogramming (Zarin et al., 2024; Zhang et al., 2020). In parallel, Rv2626c suppresses TRAF6-dependent inflammatory signaling and may additionally facilitate pathogen dissemination through induction of necrotic cell death (Kim et al., 2020; Danelishvili et al., 2016). The RD1-encoded ESX-1-associated secreted proteins EspJ (Rv3878) and EspK (Rv3879c) may contribute to permissive immunoregulatory macrophage programs during Mtb infection through the c-MAF–IL-10 axis (Tian et al., 2023). EspJ (Rv3878) has been shown to regulate mycobacterial growth and intracellular survival, and its phosphorylation by mycobacterial serine/threonine protein kinases, particularly at Ser70, influences these phenotypes in recombinant BCG and Mtb models (Singh et al., 2015). In parallel, EspK (Rv3879c)-related ESX-1 signaling has been associated with activation of the c-MAF pathway, a transcriptional program that promotes IL-10 production and immunoregulatory macrophage features. Consistently, Mtb infection induces elevated c-MAF and IL-10 expression in human CD14^hi^ monocyte-derived macrophages, whereas c-MAF silencing reduces IL-10 production and limits intracellular Mtb growth (Dhiman et al., 2011). Together, these findings suggest that EspJ (Rv3878) and EspK (Rv3879c)-associated ESX-1 signaling may contribute to c-MAF–IL-10-dependent immunoregulatory programs, thereby favoring macrophage states that are more permissive for intracellular bacterial survival. Likewise, the PPE family protein PPE36 (Rv2108) interferes with MAPK-ERK1/2 signaling and reduces production of inflammatory chemokines such as CXCL9 and CXCL10, thereby limiting local immune-cell recruitment and inflammatory activation (Gong et al., 2021).

Metabolic rewiring represents another major strategy by which Mtb reshapes macrophage functional states. Rv1987 promotes immunoregulatory, metabolically permissive features by activating PI3K–AKT–mTOR signaling and downregulating SHIP, which reduces macrophage bactericidal activity and supports intracellular mycobacterial survival (Sha et al., 2021). Rv1987 has also been shown to attenuate inflammatory responses in mouse lung and alter the lung microbiota, changes that may further favor mycobacterial persistence (Liu et al., 2023). Similarly, the DEF motif-containing protein Mce2D suppresses the ERK-HIF-1α axis and downregulates glycolysis-associated enzymes including HK2, PKM2 and LDHA, while enhancing fatty-acid oxidation and tricarboxylic acid cycle activity (Weng et al., 2023). Through coordinated suppression of glycolysis and promotion of oxidative metabolism, these virulence factors redirect macrophages away from inflammatory antimicrobial programs toward metabolically permissive states that support long-term intracellular survival of Mtb.

Mechanistically, glycolytic, oxidative, and lipid metabolic programs regulate antimicrobial effector functions rather than serving only as bioenergetic markers. In Mtb-infected macrophages, metabolic rewiring can therefore reshape nitric oxide production, inflammatory cytokine responses, mitochondrial ROS generation, phagolysosomal maturation, and xenophagy. HIF-1α-dependent glycolysis supports IFN-γ-driven macrophage activation by sustaining nitric oxide, IL-1β, and other inflammatory mediators, and aerobic glycolysis in human alveolar macrophages is required for control of intracellular bacillary replication (Gleeson et al., 2016; Braverman et al., 2016). By contrast, macrophage states dominated by OXPHOS and fatty-acid oxidation (FAO) are often associated with lower inflammatory tone and repair-biased or immunoregulatory functions, although these effects are highly context dependent. In Mtb-infected macrophages, limiting FAO can increase mitochondrial ROS, promote NOX2/NADPH oxidase recruitment to Mtb-containing phagosomes, and enhance p62/LC3-dependent xenophagy, thereby restricting intracellular bacterial growth (Chandra et al., 2020; Chai et al., 2019). Metabolic programming also intersects with lysosomal control, as antimicrobial macrophage activation favors phagosomal maturation and acidification, whereas Mtb effectors such as PtpA can exclude V-ATPase from the phagosome and block acidification, creating a non-acidified compartment permissive for bacterial survival (Wong et al., 2011).

Altered lipid metabolism further links macrophage state transitions to bacterial persistence. Mtb can recalibrate host lipid homeostasis and divert carbon flux toward lipid-body accumulation, generating foamy macrophages that provide lipid-rich niches for bacillary persistence and are frequently coupled to immune-tolerant cytokine programs (Singh et al., 2012; Holla et al., 2016). For example, tuberculous pleural effusions can drive foam-cell formation through an IL-10/STAT3/ACAT axis, directly linking lipid-droplet accumulation with immunosuppressive macrophage phenotypes (Genoula et al., 2018). However, lipid droplets should not be interpreted as uniformly pathogen-favorable, because IFN-γ/HIF-1α-driven lipid-droplet formation can support protective eicosanoid production. Thus, the consequences of lipid remodeling depend on inflammatory context, macrophage lineage, and the granuloma microenvironment (Knight et al., 2018; Genoula et al., 2020).

Mtb also interferes with intracellular antimicrobial trafficking and immune regulation through virulence-associated lipids and secreted effectors. ManLAM, a major mycobacterial cell-wall component, has been linked to impaired phagosome maturation, reduced antigen-presentation capacity and induction of immunosuppressive mediators such as IL-10 and TGF-β (Liu et al., 2017; Tang et al., 2021). In addition, ManLAM-driven release of immunoregulatory exosomes enriched in IL-4 and IL-13 can further reinforce M2-like immunoregulatory features and compromise intracellular bactericidal activity (Tang et al., 2021). These effects collectively establish an immune environment favorable for persistent infection and granuloma adaptation.

Some Mtb virulence factors additionally modulate macrophage plasticity through epigenetic and context-dependent mechanisms. Rv1899c recruits the host HDAC1-ZBTB25 transcriptional repressor complex, suppressing inflammatory mediators such as IL-12β, IL-1β and IL-6 while enhancing IL-10 expression, thereby promoting immunoregulatory macrophage states (Menon et al., 2025; Madhavan et al., 2021). ESAT-6 (Rv3875) exhibits more complex stage-dependent effects on macrophage polarization. During early infection, ESAT-6 transiently activates TLR2-NF-κB signaling and promotes NLRP3 inflammasome activation, thereby enhancing inflammatory responses. At later stages, however, it suppresses MyD88-IRAK4-dependent signaling and inhibits NF-κB and IRF activation, ultimately redirecting macrophages toward permissive anti-inflammatory states (Refai et al., 2018; Sun et al., 2024).

Taken together, Mtb virulence factors orchestrate macrophage reprogramming through convergent modulation of inflammatory signaling, metabolism, autophagy and epigenetic regulation. Rather than acting independently, these pathways form an integrated regulatory network that suppresses antimicrobial immunity while promoting immune evasion, intracellular persistence and chronic infection. Defining how these pathogen-derived effectors reshape macrophage functional states may provide important insight into host-pathogen interactions and reveal new opportunities for host-directed therapeutic intervention in tuberculosis. Their effects are also likely to vary across macrophage ontogeny and tissue niche, including alveolar, interstitial and lipid-laden foamy macrophage states (Huang et al., 2018; Pisu et al., 2021).

## 4 Host-derived and exogenous regulators of macrophage polarization during Mtb infection

With continuing advances in molecular immunology and infection biology, a growing number of host-derived regulators of macrophage polarization have been identified. These include transcription factors, receptors, cytokines and pathogen-induced host signaling molecules. Collectively, they shape macrophage fate by reconfiguring signaling cascades, transcriptional programs, metabolic circuits and organelle-associated functions. Because these regulators act on tissue-resident, recruited and lipid-laden macrophage populations, their effects should be interpreted as modulation of state trajectories rather than simple binary switching.

CYP1B1-associated signaling has been implicated in macrophage inflammatory responses in type 2 diabetes mellitus combined with tuberculosis, in part through NOD2–TRAF6-related inflammatory pathways. In this context, CYP1B1 may be better interpreted as a host regulatory node linking inflammatory and metabolic state programs rather than as a marker of a fixed M2 population (Du et al., 2025). Triggering receptor expressed on myeloid cells 2 (TREM2) is an important regulator of macrophage inflammatory responses, phagocytosis and immune evasion during Mtb infection (Dabla et al., 2022). TREM2 signaling can restrain excessive TLR–NF-κB-driven inflammation through DAP12-dependent crosstalk, thereby contributing to context-dependent immunoregulatory macrophage states (Dabla et al., 2022; Shang et al., 2024).

Granulin (GRN, also known as progranulin) can regulate the TNFR2 axis and has been linked to M2-like reparative and immunoregulatory features during Mtb infection (Zhang et al., 2024; Medler and Wajant, 2019). This GRN–TNFR2 pathway may therefore contribute to immunoregulatory macrophage states that favor bacterial persistence.

**Figure 3:**
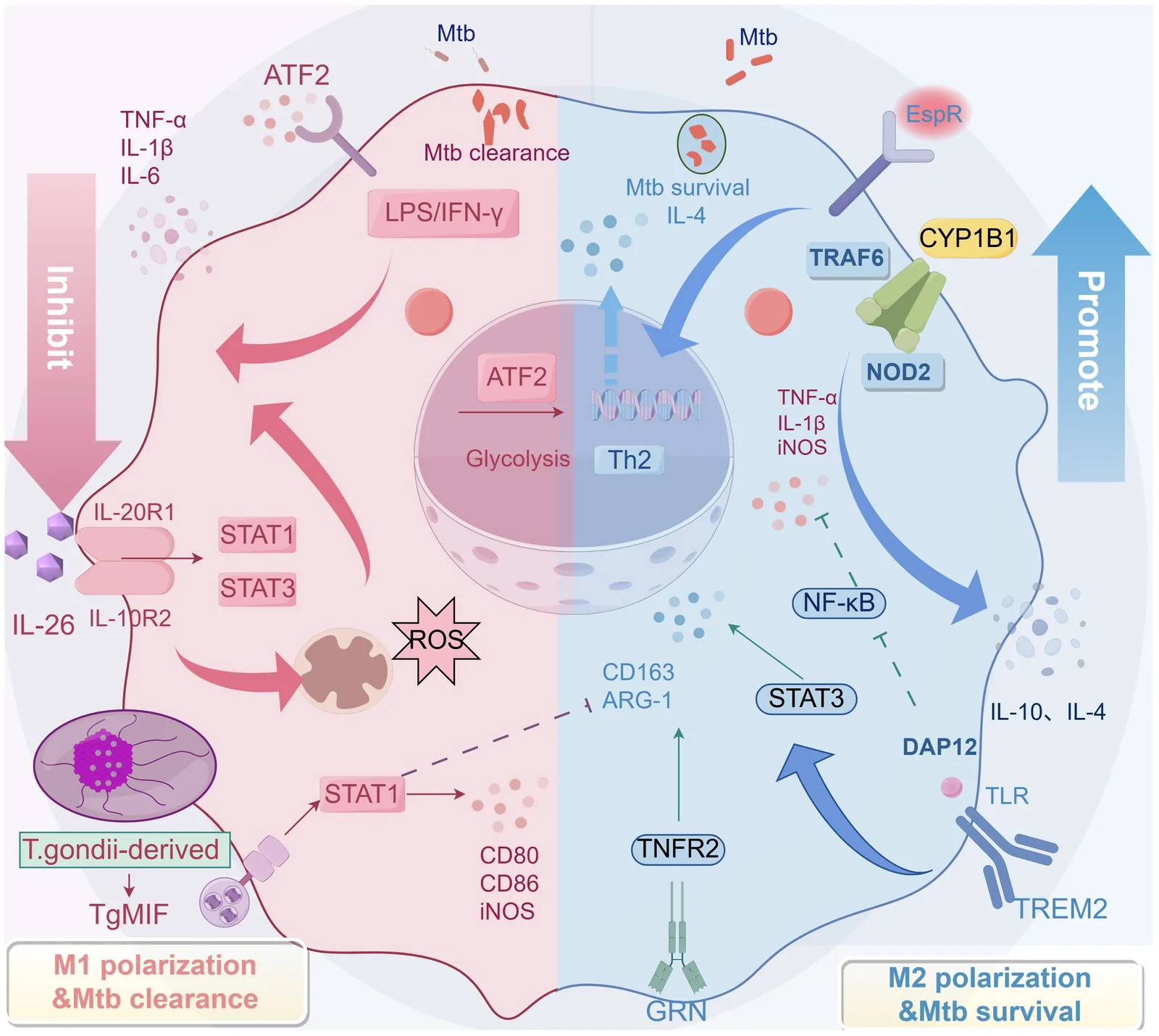
Representative host-derived and exogenous regulators of macrophage functional programs during *Mycobacterium tuberculosis* infection. The regulators shown were selected as experimentally supported examples of distinct transcriptional, cytokine, receptor-mediated, and metabolic mechanisms discussed in the main text; the figure is illustrative rather than exhaustive. Endogenous host regulators include ATF2, IL-26, CYP1B1-associated signaling, TREM2, and the GRN–TNFR2 axis. TgMIF is displayed separately as a Toxoplasma gondii-derived exogenous immunomodulatory factor and is included as a comparative example rather than as a component of the endogenous Mtb–host signaling network. ATF2 promotes antibacterial transcriptional, antigen-presentation, and glycolytic programs, whereas IL-26 enhances ROS production, autophagic activity, and intracellular mycobacterial killing. TgMIF has been associated with STAT1-dependent inflammatory and antimicrobial macrophage responses. CYP1B1 modulates NOD2–TRAF6-associated inflammatory signaling in a metabolically stressed tuberculosis model. By contrast, TREM2 can suppress TNF-α, IL-1β, and ROS production while enhancing IFN-β and IL-10, whereas the GRN–TNFR2 axis promotes immunoregulatory macrophage features. The net effects of these regulators depend on the cellular model, metabolic environment, infection stage, and tissue context and may generate overlapping inflammatory, antimicrobial, immunoregulatory, or tissue-remodeling programs rather than fixed M1- or M2-like endpoints. Created with Figdraw.

ATF2 enhances expression of M1-associated molecules, including MHC class II and IL-1β. Beyond this, it amplifies glycolytic pathways, chemokine signaling and antigen-presentation programs, thereby strengthening phagocytic and bactericidal activity. ATF2 may therefore represent a key node linking transcriptional control, immunometabolism and antimicrobial defense (Rajabalee et al., 2023). IL-26 has been reported to modulate macrophage polarization during tuberculosis and to enhance intracellular elimination of Mtb through IL-20R1/IL-10R2-associated STAT1/STAT3 signaling (Huang et al., 2024). Toxoplasma gondii-derived macrophage migration inhibitory factor (TgMIF), a structural homologue of mammalian MIF, serves as an exogenous effector with significant immunomodulatory capacity. Its activity relies on specific interaction with STAT1, through which the TgMIF–STAT1 axis supports acquisition of inflammatory antimicrobial features. This reprogramming improves antigen presentation and cytotoxicity and may involve reactive nitrogen species. TgMIF also inhibits M2-associated molecules such as CD163, CD206 and arginase 1, thereby weakening an immunometabolic milieu conducive to Mtb survival (Sommerville et al., 2013; Zhou et al., 2023; Yoon et al., 2024). These host-derived and exogenous regulators are summarized in **Figure 3**.

Overall, these host-derived and exogenous regulators shape macrophage polarization through convergent but mechanistically distinct routes. By modulating inflammatory signaling, transcriptional circuitry and metabolic state, they critically influence the balance between antimicrobial defense and immune tolerance during Mtb infection.

## 5 Epigenetic regulation of macrophage polarization during Mtb infection

Mtb is an intracellular pathogen that persists inside host macrophages by altering their activity. It interferes with the continuum of inflammatory, antimicrobial, reparative and immunoregulatory macrophage programs. Rather than producing a uniform shift from M1-like to M2-like macrophages, these changes generate mixed or transitional states with variable antimicrobial capacity, thereby supporting prolonged intracellular survival. Epigenetic reprogramming is a key driver of these changes. Mtb rewires macrophage transcriptional profiles by affecting histone modifications, DNA methylation, and non-coding RNA networks, often alongside metabolic shifts, to facilitate its persistence and replication (Kathirvel and Mahadevan, 2016; Zhang et al., 2020; Kim et al., 2020).

### 5.1 Histone acetylation reprograms macrophage inflammatory responses

Mtb actively reshapes macrophage transcriptional programs through histone acetylation-dependent mechanisms that can favor anti-inflammatory polarization. The secreted effector Rv3423.1 translocates into the macrophage nucleus and functions as a histone acetyltransferase, catalyzing acetylation of histone H3 at lysines 9 and 14 (H3K9/14ac) at the IL-10 promoter (Fatima et al., 2022; Jose et al., 2016). This modification facilitates recruitment of RNA polymerase II and sustains IL-10 transcription, thereby suppressing pro-inflammatory cytokine production, antigen presentation and autophagy, ultimately promoting M2-like immunoregulatory features (Jose et al., 2016; Fatima et al., 2022). Similarly, the Mtb EIS gene inhibits macrophage autophagy by upregulating IL-10 through increased histone H3 acetylation (Duan et al., 2016).

In parallel, Mtb suppresses inflammatory gene expression through host histone deacetylases. Mtb infection induces HDAC1 and HDAC3 expression and promotes their recruitment to promoters of key pro-inflammatory genes, including TNF, IL12B and NOS2 (Grabiec and Potempa, 2018; Chandran et al., 2015). This coordinated activation of anti-inflammatory acetylation programs alongside deacetylation-mediated repression of antimicrobial genes enables Mtb to remodel macrophage chromatin landscapes toward an immunoregulatory state permissive for intracellular persistence.

### 5.2 Histone methylation couples epigenetic regulation to metabolic polarization

Beyond histone acetylation, Mtb exploits histone methylation to coordinate immune suppression with metabolic rewiring. Mtb-mediated suppression of CDT2 stabilizes the histone methyltransferase SET8 through a miR-30e-3p-dependent pathway, resulting in accumulation of H4K20me1 at promoters of antioxidant and oxidative metabolic genes, including NQO1 and TRXR1 (Abbas et al., 2010; Serrano et al., 2013; Madden et al., 2022). Upregulation of these pathways stabilizes PGC-1α, a master regulator of mitochondrial biogenesis and fatty acid oxidation, thereby shifting macrophages along a glycolytic-inflammatory to oxidative-immunoregulatory trajectory (Singh et al., 2017; Li et al., 2016).

Mtb also promotes transcriptional silencing of inflammatory genes through H3K27 trimethylation, a canonical PRC2-associated repressive mark (Margueron and Reinberg, 2011). By suppressing the histone demethylase KDM6B (JMJD3), Mtb sustains H3K27me3 accumulation at promoters of key M1-associated genes, including IL-12, TNF and NOS2 (Satoh et al., 2010; Holla et al., 2016; Sengupta et al., 2021). This repressive chromatin configuration limits recruitment of transcription factors such as NF-κB and STAT1, thereby attenuating antimicrobial inflammatory responses. In addition, ESAT-6 has been reported to dysregulate host methyltransferase activity and alter H3K4 methylation at IFN-γ-responsive loci, impairing IFN-γ-dependent transcriptional programs and weakening classical macrophage activation (Kumar et al., 2010; Kumar P. et al., 2012).

**Figure 4:**
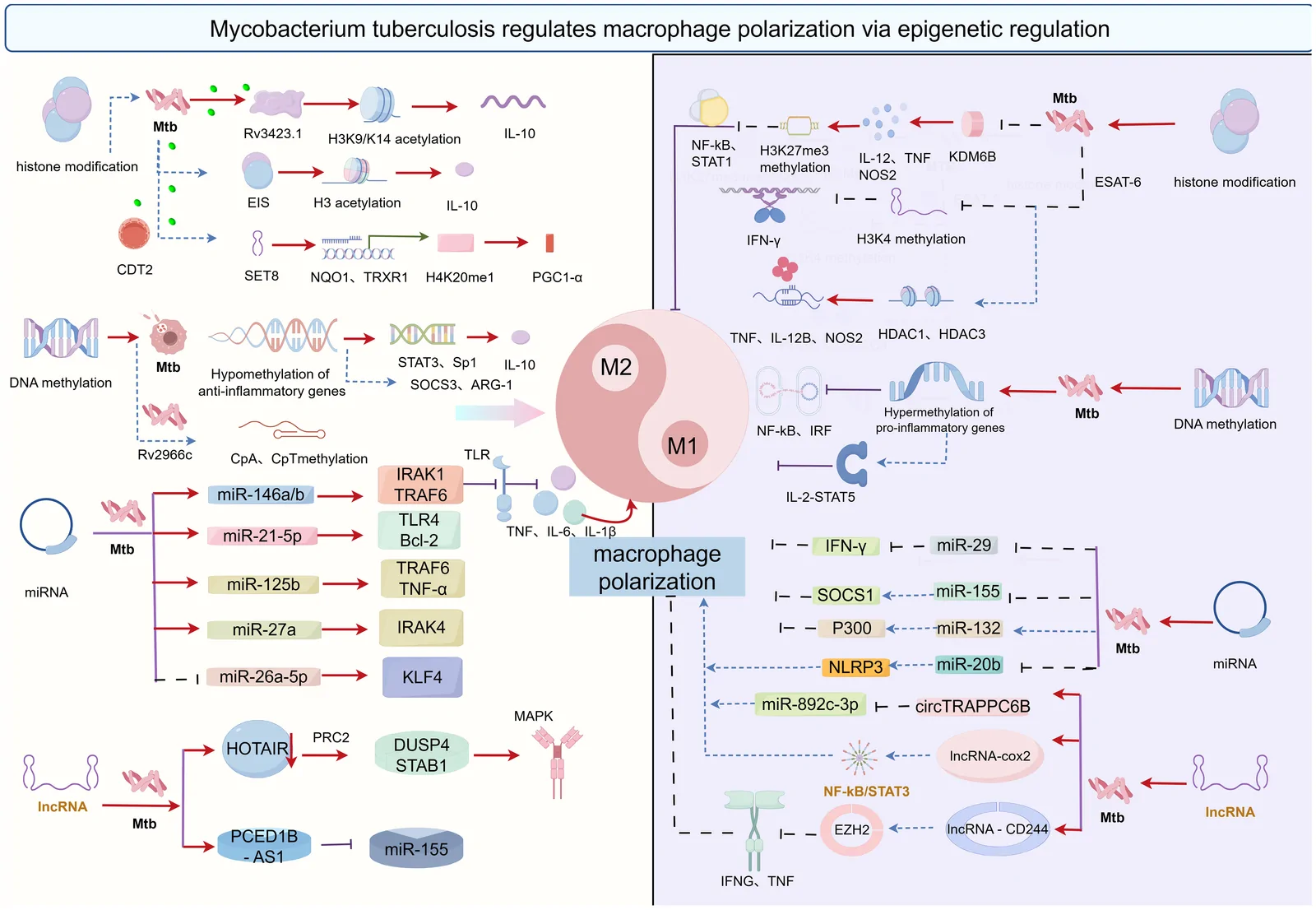
Mtb reprograms macrophage polarization through coordinated epigenetic mechanisms. The schematic summarizes how *Mycobacterium tuberculosis* reshapes macrophage polarization through coordinated epigenetic mechanisms, including histone modifications, DNA methylation and non-coding RNA networks. The left panel shows regulators mainly associated with immunoregulatory, metabolic or M2-like macrophage programs, including histone-modification-related regulators Rv3423.1, EIS and CDT2–SET8; DNA methylation-related regulators together with Rv2966c-mediated CpA/CpT methylation; and non-coding RNA regulators including miR-146a/b, miR-21-5p, miR-125b, miR-27a, miR-26a-5p, HOTAIR and PCED1B-AS1. The right panel illustrates epigenetic and non-coding RNA circuits that modulate inflammatory and antimicrobial macrophage programs, including KDM6B-dependent H3K27me3 demethylation, ESAT-6-associated histone methylation, HDAC1/HDAC3-dependent repression, DNA hypermethylation, and regulation by miR-29, miR-155, miR-132, miR-20b, circTRAPPC6B, lncRNA-COX2 and lncRNA-CD244. Together, these mechanisms indicate that Mtb does not drive a simple M1/M2 switch, but instead remodels macrophage polarization through interconnected chromatin, transcriptional and non-coding RNA pathways that influence inflammatory, antimicrobial, metabolic and immunoregulatory states. Created with Figdraw.

Collectively, these findings suggest that Mtb integrates histone methylation with metabolic adaptation to stabilize macrophages in an immunoregulatory state that supports bacterial persistence (**Figure 4**). Such chromatin-metabolic coupling may help explain why foamy or hybrid macrophage states can persist within granulomas despite ongoing inflammatory cues.

### 5.3 DNA methylation establishes stable immunosuppressive transcriptional states

DNA methylation represents a more stable layer of epigenetic regulation through which Mtb reinforces long-term immune tolerance. In Mtb-infected macrophages, hypomethylation of CpG islands within promoters and enhancers of anti-inflammatory genes such as IL-10 enhances chromatin accessibility and facilitates recruitment of transcription factors including STAT3 and Sp1, thereby sustaining IL-10 expression (Zheng et al., 2016, 2020). Similar hypomethylation patterns have been observed in other M2-associated genes, including SOCS3 and ARG-1, collectively contributing to an immunosuppressive microenvironment favorable for intracellular survival (Dinardo et al., 2020).

Conversely, Mtb promotes hypermethylation of promoters associated with pro-inflammatory and IFN-responsive pathways, including TNF, IFNG and STAT1, thereby restricting NF-κB- and IRF-dependent transcriptional activation (Thomas et al., 2024). Hypermethylation of components within the IL-2–STAT5 axis may further impair crosstalk between innate and adaptive immunity (Dinardo et al., 2020). Importantly, Mtb also exerts direct epigenetic control through secreted bacterial effectors. The mycobacterial protein Rv1988 alters histone H3 arginine methylation to suppress host gene transcription (Yaseen et al., 2015), whereas Rv2966c functions as a non-canonical DNA methyltransferase capable of catalyzing cytosine methylation at CpA and CpT motifs (Yaseen et al., 2015; Khadela et al., 2022; Sharma et al., 2016). These pathogen-driven epigenetic modifications repress genes involved in autophagy, antigen processing and antimicrobial defense, highlighting a mechanism of cross-kingdom epigenetic regulation that facilitates persistent infection (Sharma et al., 2015).

## 6 Non-coding RNA networks in macrophage reprogramming

Beyond covalent modifications of DNA and histones, Mtb reshapes the host non-coding RNA landscape to establish a dynamic regulatory network operating at both transcriptional and posttranscriptional levels. MicroRNAs and long non-coding RNAs are central components of this network. By targeting key nodes within immune signaling pathways, they coordinately regulate macrophage polarization, autophagy and apoptosis, thereby creating a finely balanced system at the interface between host defense and bacterial immune evasion.

### 6.1 MicroRNA networks in macrophage reprogramming

MicroRNAs are major post-transcriptional regulators and have an important role in shaping macrophage plasticity during Mtb infection. miR-146a and miR-146b are robustly upregulated after infection (Looney et al., 2021; Ghorpade et al., 2013). These microRNAs suppress TLR signaling by targeting IRAK1 and TRAF6, thereby reducing TNF, IL-6 and IL-1β production and creating a low-inflammatory niche that supports Mtb persistence (Hou et al., 2009). miR-21-5p targets TLR4 and also modulates genes such as BCL2, thereby influencing apoptosis and metabolic programming. Through these effects, it suppresses the aerobic glycolysis characteristic of M1-like macrophages and promotes a shift toward an oxidative phosphorylation-dependent M2-like state (Sheedy et al., 2010). Likewise, miR-125b and miR-27a target TRAF6/TNF and IRAK4, respectively, thereby imposing coordinated inhibition at distinct levels of the TLR axis (Niu et al., 2018; Wang et al., 2017). In murine macrophages, Mtb downregulates miR-26a-5p expression, which suppresses translation of Kruppel-like factor 4 (KLF4) (Sahu et al., 2017). Loss of this restraint facilitates KLF4-dependent skewing toward an M2-like state.

Complementing these immunosuppressive mechanisms, Mtb also appears to weaken host defense by perturbing protective microRNAs. The miR-29 participates in regulation of IFN-γ expression and Th1 immunity, and its dysregulation can disrupt immune feedback control and impair macrophage activation (Ma et al., 2011). In contrast, miR-155 strengthens inflammatory responses and promotes autophagy through suppression of SOCS1, thereby contributing to host protection (Kumar R et al., 2012; Holla et al., 2014). In human macrophages, miR-26a and miR-132 can be upregulated during Mtb infection and cooperatively target the transcriptional co-activator p300 (Ni et al., 2014). Suppression of p300 not only remodels chromatin acetylation but also weakens coactivation of transcription factors such as STAT1, thereby attenuating IFN-γ-driven antimicrobial responses and suppressing M1-like polarization. The host can also amplify inflammatory responses through selected microRNAs. miR-20b is downregulated during Mtb infection, relieving repression of NLRP3, promoting inflammasome assembly and activating the NLRP3-IL-1β-caspase-1 axis, which ultimately drives M1-like inflammatory responses (Lou et al., 2017).

### 6.2 lncRNA regulatory networks: linking epigenetic remodeling to transcriptional control

Long non-coding RNAs also contribute to Mtb-induced immune reprogramming (Kundu and Basu, 2021). Specifically, they can function as molecular scaffolds, chromatin regulators or competitive endogenous RNAs. Through these roles, they link epigenetic remodeling to signal transduction. Some long non-coding RNAs suppress inflammatory gene expression by recruiting chromatin-modifying complexes. lncRNA-CD244 can bind EZH2, a core component of polycomb repressive complex 2, recruiting the complex to the IFNG and TNF loci. This process promotes deposition of the repressive H3K27me3 mark and silences these key inflammatory genes (Wang et al., 2015). In parallel, some host lncRNAs reinforce inflammatory responses and antimicrobial defense. LncRNA-COX2 is an early-response transcript downstream of TLR signaling and is markedly upregulated in Mtb-infected macrophages. It promotes pro-inflammatory mediators such as TNF and IL-6 expression via activation of NF-κB and STAT3, driving macrophages toward a bactericidal M1-like state (Li et al., 2020). Similarly, HOTAIR, an important PRC2-recruiting lncRNA, is downregulated during Mtb infection. This change may relieve repression of genes such as DUSP4 and SATB1, thereby reconfiguring MAPK signaling and chromatin architecture and establishing a cellular state more permissive for bacterial persistence (Subuddhi et al., 2020).

Long non-coding RNAs can also regulate polarization through microRNA-sponging mechanisms. PCED1B-AS1 is upregulated during Mtb infection and functions as a ceRNA for miR-155, thereby reducing miR-155 activity, suppressing miR-155-dependent autophagy and inflammatory responses and promoting intracellular proliferation of Mtb (Li et al., 2019). Similarly, circTRAPPC6B modulates inflammatory cytokine expression by sponging miR-892c-3p. Under physiological antimicrobial conditions, circTRAPPC6B restrains miR-892c-3p and promotes IL-6 and IL-1β expression, thereby favoring M1-like polarization. During Mtb infection, however, circTRAPPC6B is downregulated, allowing miR-892c-3p to accumulate and suppress IL-6 and IL-1β, which ultimately favors M2-like polarization and immune evasion (Peng et al., 2023; Luo et al., 2021).

Taken together, Mtb systematically reprograms host microRNA and long non-coding RNA networks to reshape inflammatory signaling, epigenetic states and metabolic programs at multiple levels. These coordinated effects, which favor immunosuppressive or reparative macrophage programs within a broader activation continuum and support persistent intracellular infection, are summarized in **Figure 4**.

## 7 Therapeutic implications

### 7.1 Macrophage polarization in granuloma formation

Granuloma formation is one of the defining pathological hallmarks of Mtb infection. At its core, the granuloma is a highly organized inflammatory microenvironment assembled by the host to contain pathogen dissemination and composed of multiple immune-cell populations acting in concert (Wu et al., 2021; Silva Miranda et al., 2012; Pagán and Ramakrishnan, 2014). Dynamic shifts in macrophage polarization are therefore increasingly viewed as major determinants of granuloma evolution and infection outcome (Wu et al., 2021; Cyktor et al., 2013). Mtb initially induces bone marrow-derived macrophages to acquire inflammatory antimicrobial features. This phase is characterized by increased expression of iNOS, TNF and CXCL11, thereby enhancing antimycobacterial activity (Wu et al., 2021). In parallel, M2-associated markers such as CD206 and CCL17 are downregulated, indicating that the initial host response is dominated by inflammatory defense (Shang et al., 2024). As infection persists and the local immune milieu is remodeled, however, macrophages can accumulate reparative, immunoregulatory, foamy or hypoxia-adapted features that may favor Mtb persistence by upregulating anti-apoptotic molecules, suppressing lysosomal maturation and secreting immunosuppressive mediators such as IL-10 and TGF-β. This structural remodeling promotes central hypoxia, caseous necrosis, and peripheral fibrosis, thereby establishing a local environment favorable for long-term pathogen survival (Medler and Wajant, 2019). Studies of human tuberculosis lesions demonstrate that macrophage polarization within granulomas is highly heterogeneous rather than strictly binary. Granuloma-associated macrophages frequently exhibit iNOS^low^ CD206^high^ or mixed inflammatory reparative phenotypes, reflecting continuous adaptation to local cytokine, metabolic and hypoxic cues (Mattila et al., 2013; Qiu et al., 2024). In chronic lesions, fibrotic and calcified granulomas are often enriched in CD163^high^ reparative macrophages with reduced inflammatory activity, consistent with tissue-remodeling programs. Functionally, excessive inflammatory activation may promote tissue destruction, whereas dominant reparative states stabilize granuloma structure but facilitate bacterial persistence and latent infection. Thus, macrophage polarization critically shapes granuloma fate and tuberculosis progression.

This temporal pattern should not be interpreted as a uniform linear conversion from M1-like to M2-like programs. Instead, granuloma maturation likely reflects changing proportions and spatial localization of multiple macrophage states, including inflammatory, IFN-responsive, lipid-laden, hypoxia-adapted and reparative populations, whose functional impact depends on lesion stage and microanatomical niche. At the tissue level, these polarization features are distributed across distinct macrophage lineages rather than across a single uniform population. Lung-resident alveolar macrophages and interstitial/monocyte-derived macrophages differ in ontogeny, metabolism and permissiveness to Mtb: bacilli in alveolar macrophages show lower stress and higher replication, whereas interstitial macrophages display glycolytic inflammatory programs that more effectively restrict bacterial growth (Huang et al., 2018). Reporter-based scRNA-seq further resolved pro-inflammatory alveolar macrophage clusters and multiple interstitial macrophage subpopulations with distinct bacterial phenotypes (Pisu et al., 2021). In parallel, foamy macrophages represent lipid-remodeled, tissue-adapted states within granulomas rather than a simple M2 endpoint, linking lipid-droplet accumulation, IL-10/STAT3 signaling and epigenetic remodeling to persistence (Genoula et al., 2018; Holla et al., 2016).

### 7.2 Host-directed therapeutic strategies targeting macrophage polarization

Host-directed therapies (HDTs) aim to improve tuberculosis treatment by reprogramming macrophage functional states to enhance antimicrobial immunity while limiting tissue-destructive inflammation (Jeong et al., 2022). Because Mtb promotes immunoregulatory macrophage programs through coordinated manipulation of host signaling, metabolism and epigenetic pathways, reversal of these permissive states has emerged as a promising therapeutic strategy (Mizutani et al., 2023).

Several signaling pathways involved in macrophage polarization have therefore become attractive therapeutic targets. Inhibition of the PI3K–AKT axis can suppress IL-10 production and restore antimicrobial activity by relieving inhibition of autophagy and inflammatory signaling (Bouzeyen et al., 2019; Li et al., 2025). Conversely, activation of p38 MAPK promotes inflammatory macrophage programs and enhances intracellular mycobacterial clearance (Pahuja et al., 2023; Kumari et al., 2023; Singh et al., 2022). In addition to kinase-centered strategies, ocaglates provide a mechanistically distinct HDT example by acting at the level of translational control. These compounds inhibit eIF4A, an RNA helicase within the eIF4F translation-initiation complex, and thereby selectively suppress translation of eIF4A-sensitive mRNAs rather than producing uniform global translational arrest. In macrophages, the immunomodulatory activity of active rocaglate derivatives correlates with their translational-inhibitory potency and is associated with marked suppression of Myc protein despite maintained or increased Myc mRNA expression, indicating preferential inhibition of Myc-associated translational programs. Rocaglate treatment also induces a stress-response-like remodeling state characterized by activation of p38 MAPK and increased ATF4, ATF3 and SQSTM1/p62, which can sensitize macrophages to low-dose IFN-γ and enhance IRF1/IRF5-associated antimicrobial programs. In parallel, rocaglates suppress IL-4- and CSF1-associated anabolic or alternative-activation pathways, including ERK/STAT6 and Myc-dependent regulatory modules. Through this combined inhibition of eIF4A-sensitive Myc-associated translation and activation of stress-adaptive antimicrobial networks, rocaglates promote phagosome-lysosome fusion and improve macrophage control of intracellular mycobacteria (Chatterjee et al., 2021; Wolfe et al., 2014; Iwasaki et al., 2019). Additional approaches aim to restore macrophage responsiveness to IFN-γ, enhance autophagy or strengthen crosstalk between innate and adaptive immunity.

**Figure 5:**
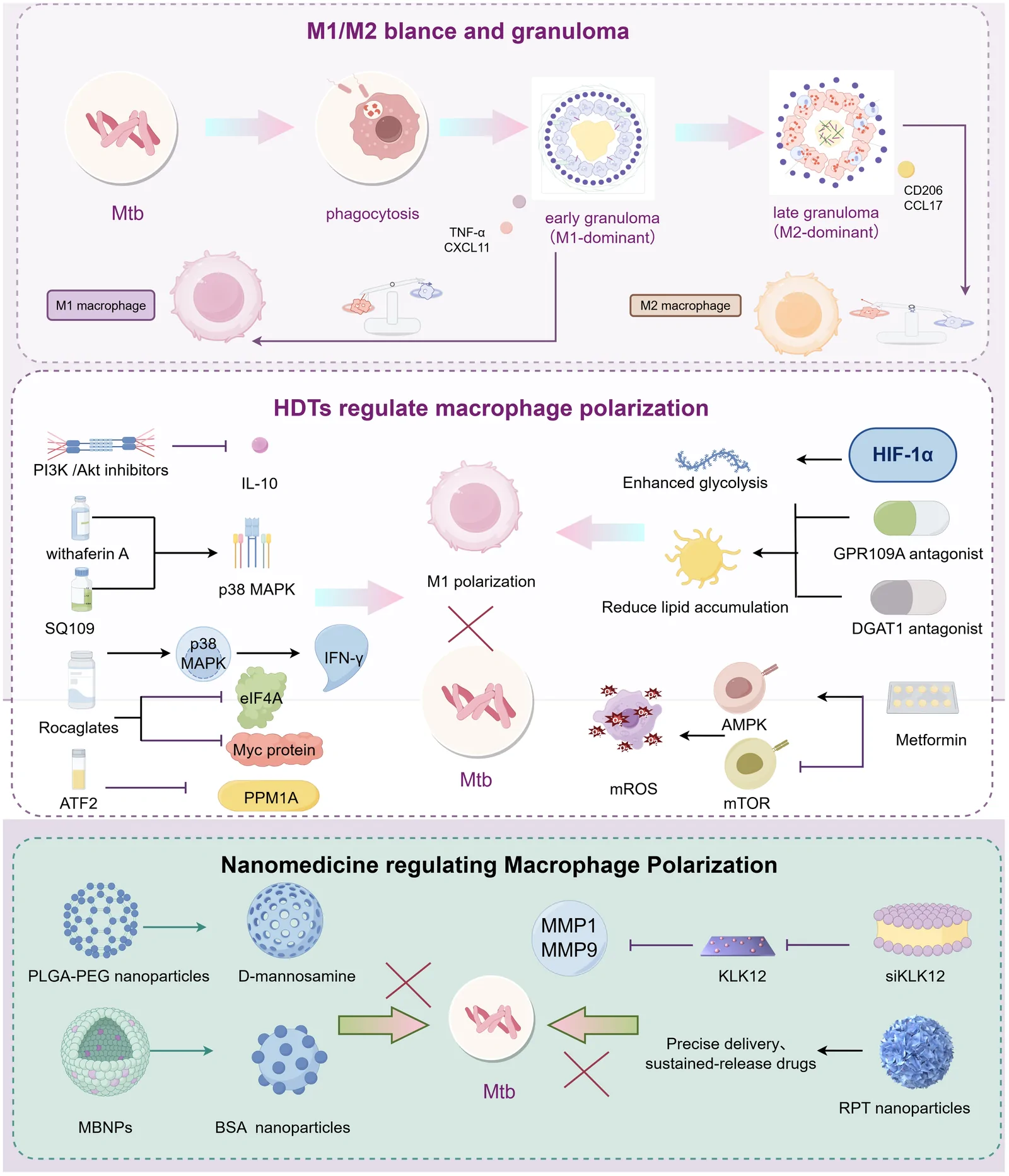
Macrophage polarization links granuloma evolution with host-directed and nanomedicine-based interventions in tuberculosis. The schematic integrates three related concepts. First, granuloma evolution is associated with changing macrophage programs: early lesions are enriched for inflammatory and antimicrobial signals, whereas chronic or late lesions can accumulate reparative, immunoregulatory and foamy macrophage states that favor persistence. Second, HDT nodes such as PI3K/Akt–IL-10, p38 MAPK, Myc/eIF4A-sensitive translation, ATF2–PPM1A, HIF-1α-glycolysis, GPR109A/DGAT1-dependent lipid accumulation and metformin–AMPK/mTOR/mROS illustrate signaling and metabolic routes for restoring antimicrobial competence while limiting inflammatory injury. Third, the nanomedicine module highlights mannose/mannosamine-functionalized CD206-targeted carriers for rifapentine or other anti-TB payloads, autophagy-modulating nanomaterials and KLK12-targeting siRNA nanoparticles designed to reduce MMP-associated matrix degradation and granuloma disruption. PLGA-PEG/D-mannosamine rifapentine nanoparticles and mannose-modified BSA nanoparticles carrying siKLK12 are shown as representative delivery platforms. This figure is a simplified conceptual model, not a universal linear polarization trajectory; *in vivo* granulomas contain heterogeneous macrophage populations with spatially and temporally overlapping inflammatory, foamy, reparative and immunoregulatory states. Created with Figdraw.

These pathway-centered strategies illustrate how macrophage-directed HDTs can rebalance interconnected signaling, transcriptional, translational and metabolic programs rather than act through a single pro-inflammatory switch. PI3K–Akt inhibition may attenuate IL-10-associated immunoregulatory signaling, whereas withaferin A and SQ109 converge on p38 MAPK signaling to strengthen antimicrobial inflammatory programs. Rocaglates provide a distinct translational-control mechanism by inhibiting eIF4A-dependent initiation and reshaping Myc-sensitive regulatory programs, thereby enhancing IFN-γ-dependent antimicrobial activation. In parallel, ATF2 supports macrophage differentiation, glycolytic remodeling and antibacterial responses, whereas PPM1A acts as a phosphatase checkpoint that can blunt cytokine and chemokine secretion, chemotaxis and phagocytosis during Mtb infection. Together, these examples support HDT strategies that improve antimicrobial competence while limiting inflammatory injury, as summarized in **Figure 5** (Bouzeyen et al., 2019; Kumari et al., 2023; Singh et al., 2022; Chatterjee et al., 2021; Rajabalee et al., 2023; Sun et al., 2016).

Metabolic rewiring also represents a major focus of HDT development. Enhancement of HIF-1α-dependent glycolysis can reinforce antimicrobial macrophage functions (Shi et al., 2015; Gleeson et al., 2016; Li et al., 2021), whereas lipid-directed strategies, including GPR109A antagonism and DGAT1 inhibition, may reduce triglyceride or lipid-body accumulation in foamy macrophage states and limit metabolically permissive niches for Mtb persistence (Singh et al., 2012; Dawa et al., 2021). However, these strategies require context-dependent evaluation because fatty-acid oxidation and lipid droplets can have divergent effects across macrophage lineages and inflammatory settings; indiscriminate suppression of lipid droplets could remove protective eicosanoid-producing lipid droplets in IFN-γ-activated macrophages (Chandra et al., 2020; Genoula et al., 2020; Knight et al., 2018). Thus, metabolic HDTs should be assessed by restoration of downstream antimicrobial effector functions, including ROS production, phagolysosomal control and xenophagy, rather than by changes in metabolic flux alone.

Among current HDT candidates, metformin is particularly notable because it integrates multiple immunometabolic mechanisms relevant to macrophage polarization (Singhal et al., 2014). Through activation of AMPK and suppression of mTOR signaling, metformin promotes autophagy, enhances mitochondrial ROS production and limits excessive inflammatory responses (Jeong et al., 2022; O'Neill and Hardie, 2013; West et al., 2011). In addition, metformin may strengthen adaptive immunity by promoting expansion of Mtb-specific CD8^+^ T cells (Sutter et al., 2024; Pearce et al., 2009; Commandeur et al., 2011).

Collectively, these findings identify macrophage polarization as a central therapeutic axis in tuberculosis. Future HDT strategies will likely require precise modulation of macrophage functional states to optimize bacterial clearance while minimizing chronic inflammation, tissue necrosis, lung cavitation and granuloma-associated pathology. This balance is essential because excessive or unresolved inflammatory polarization can amplify pro-inflammatory mediator-driven tissue injury and matrix remodeling; therefore, successful HDTs should restore coordinated antimicrobial and regulatory functions rather than simply enhance pro-inflammatory responses (Ahmad et al., 2022; Tiwari and Martineau, 2023).

### 7.3 Nanocarrier-based targeting of macrophages in drug-resistant tuberculosis

Integration of nanotechnology with host-directed therapy has emerged as a promising strategy for multidrug-resistant tuberculosis by improving intracellular drug delivery and enabling selective modulation of macrophage functional states (Bourguignon et al., 2023; Liu et al., 2024; Luan et al., 2025). Because CD206 expression is enriched in selected Mtb-infected, reparative or immunoregulatory macrophage states, mannose-functionalized nanocarriers can preferentially target these macrophage populations while minimizing off-target effects (Wang et al., 2024).CD206-targeted nanoparticles have been developed to deliver both antimicrobial agents and gene-based therapeutics directly into macrophages. In addition to enhancing intracellular accumulation and bactericidal efficacy of conventional anti-tubercular drugs, these systems can selectively deliver siRNAs that suppress pathological immunoregulatory pathways (Luan et al., 2025; Wang et al., 2024; Paurević et al., 2024).

Macrophage-targeted nanocarriers can support tuberculosis therapy through several complementary mechanisms. Mannose- or mannosamine-functionalized carriers exploit CD206/mannose receptor-mediated uptake to enhance macrophage accumulation of rifapentine or other anti-tubercular payloads and prolong local drug release (Bourguignon et al., 2023; Luan et al., 2025; Paurević et al., 2024). In parallel, functional nanomaterials may act as host-directed interventions by promoting autophagy or phagolysosomal maturation, thereby reinforcing intracellular Mtb clearance (Liu et al., 2024). Nanoparticle-based gene delivery provides an additional strategy by silencing pathogenic host targets such as KLK12 in CD206-enriched immunoregulatory or tissue-remodeling macrophage states, reducing MMP-1/MMP-9-associated matrix degradation, granuloma disruption, and bacterial dissemination (Wang et al., 2024, 2022).

Representative examples include D-mannosamine-functionalized PLGA-PEG nanoparticles loaded with rifapentine, which are designed to improve macrophage uptake, prolong drug release, and enhance intracellular clearance of Mtb (Luan et al., 2025). Mannose-modified BSA nanoparticles carrying siKLK12 provide another example of CD206-directed delivery; by suppressing KLK12-driven MMP-1/MMP-9 expression, these nanoparticles may preserve collagen fibers and limit disease progression in tuberculosis and drug-resistant tuberculosis models (Wang et al., 2024). These examples illustrate how nanocarrier design can combine antimicrobial delivery with selective modulation of macrophage states and granuloma-associated tissue remodeling.

Viewed in this way, macrophage-targeted nanocarriers extend beyond antibiotic pharmacokinetics by coupling intracellular drug or siRNA delivery with selective remodeling of macrophage states and granuloma-associated pathology. By integrating CD206/mannose receptor-mediated uptake, autophagy or phagolysosomal modulation and KLK12–MMP-associated matrix preservation, these platforms may improve bacterial clearance while limiting tissue-destructive inflammation. **Figure 5** places this nanomedicine concept alongside the signaling and metabolic HDT modules discussed above.

Despite their therapeutic promise, aerosolized or nanoparticle-based HDTs against virulent Mtb require rigorous biosafety evaluation before translational application. Studies involving aerosol generation, nebulizers, pulmonary delivery devices, or infected animal exposure should be performed under appropriate BSL-3 containment after institutional risk assessment and protocol approval. Key safeguards include trained personnel, appropriate respiratory protection and PPE, directional airflow, HEPA-filtered exhaust, validated decontamination procedures, and sealed exposure or certified containment systems to minimize uncontrolled aerosol release. Experimental workflows should also include procedures for nanomaterial-contaminated waste and equipment decontamination. Whenever possible, attenuated strains, non-virulent surrogates, or noninfectious particle models should be used for preliminary optimization before testing virulent Mtb (Meechan and Potts, 2020; World Health Organization, 2020).

## 8 Discussion and perspectives

Macrophage polarization is a central determinant of host–pathogen interactions in tuberculosis. Rather than representing a rigid M1/M2 dichotomy, macrophage activation during Mtb infection constitutes a dynamic and heterogeneous spectrum shaped by mycobacterial virulence factors, host signaling networks, metabolic adaptation and epigenetic reprogramming (Marakalala et al., 2018). Through coordinated manipulation of these interconnected pathways, Mtb establishes permissive macrophage states that support intracellular survival, granuloma remodeling and chronic infection, whereas host immune mechanisms attempt to restore antimicrobial activity while limiting inflammatory injury.

Increasing evidence suggests that macrophage functional states are spatially and temporally regulated within granulomatous lesions, highlighting the limitations of conventional binary polarization models. A deeper understanding of macrophage plasticity and granuloma ecology may therefore provide important insight into disease heterogeneity, treatment response and tuberculosis progression. These advances also position macrophage reprogramming as a promising therapeutic axis for host-directed interventions, including immunometabolic modulation and macrophage-targeted nanomedicine strategies designed to enhance bacterial clearance while limiting tissue-destructive inflammation.

Recent single-cell transcriptomic, spatial transcriptomic and multi-omic approaches now provide the tools to connect macrophage ontogeny, lesion-specific niches, chromatin accessibility, metabolic programming and bacterial fitness at cellular and spatial resolution (Pisu et al., 2021; Qiu et al., 2024). These studies support a continuum model in which transitional, hybrid, foamy, interferon-responsive and tissue-adapted macrophage states are shaped by bacterial burden, cytokine gradients, metabolic stress, hypoxia and local tissue architecture. By mapping which states associate with bacterial control, tissue damage or treatment response, these technologies can guide context-specific HDT prioritization and biomarker development.

Important limitations nevertheless remain. Current datasets differ in species, lesion stage, tissue sampling, infection burden and analytical strategy, which complicates the definition of reproducible macrophage-state signatures. Future work should therefore integrate transcriptional, epigenetic, metabolic, spatial and functional perturbation readouts with bacterial burden and treatment-response measurements, enabling protective macrophage programs to be distinguished from permissive, tissue-remodeling or foamy states.

At the granuloma level, macrophage-state biology must also be interpreted in spatial context. Distinct microanatomical regions, including the necrotic core, macrophage-rich cellular cuff and fibrotic rim, differ in oxygen tension, nutrient availability, lipid metabolites and immune-cell interactions. Accordingly, alveolar and interstitial/monocyte-derived macrophages, together with lipid-remodeled foamy macrophage states, may occupy overlapping but functionally distinct niches with different metabolic biases and antimicrobial capacities (Huang et al., 2018; Pisu et al., 2021; Qiu et al., 2024). These local conditions may support different macrophage programs, ranging from inflammatory and bactericidal states to lipid-rich, reparative or immunoregulatory phenotypes (Mattila et al., 2013; Pagán and Ramakrishnan, 2014; Qiu et al., 2024). Although inflammatory–antimicrobial macrophage programs are essential for Mtb control, their magnitude and duration must be tightly regulated. TNF, IL-1, reactive oxygen species, and reactive nitrogen species contribute to macrophage activation and granuloma organization, but excessive or sustained inflammatory signaling can promote macrophage necrosis, neutrophil recruitment, protease and matrix metalloproteinase activity, and extracellular-matrix destruction, thereby contributing to caseous necrosis and pulmonary cavitation (Yuk et al., 2024). Tissue-destructive effects have also been associated with M1-related inflammatory cytokines in spinal tuberculosis, although these findings reflect a distinct anatomical microenvironment and should not be directly extrapolated to pulmonary cavitation (Wang et al., 2020). Therefore, successful HDTs should preserve antimicrobial activity while restraining excessive inflammation and tissue injury, rather than simply enhancing M1-like polarization. Such spatial organization may help explain why some granulomas contain or restrict infection, whereas others become permissive niches for bacterial persistence or reactivation. A better understanding of granuloma topology will therefore be essential for designing HDTs that remodel pathogenic macrophage states without destabilizing protective granuloma architecture (Russell et al., 2025).

Clinical translation of host-directed therapies targeting macrophage polarization remains limited, despite their therapeutic promise. Metformin, statins and rocaglates have shown potential to enhance antimicrobial macrophage functions through pathways related to AMPK activation, autophagy, immunometabolic remodeling and inflammatory regulation (Singhal et al., 2014; Chatterjee et al., 2021; Sutter et al., 2024). However, much of the evidence for these agents still derives from *in vitro* studies, animal models or observational clinical data. Statins, for example, can promote autophagy and phagosome maturation and improve anti-tuberculosis treatment responses in mouse models, but their efficacy, safety, timing and patient-selection criteria in human tuberculosis require further validation (Parihar et al., 2014; Dutta et al., 2020). Future trials should incorporate immune and metabolic biomarkers to identify patient subgroups most likely to benefit from macrophage-targeted HDTs.

Nanomedicine-based strategies offer a promising approach to selectively deliver antimicrobial drugs, siRNAs or immunomodulatory agents into infected macrophages. Nevertheless, most macrophage-targeted nanocarrier studies in tuberculosis remain at the preclinical stage, especially in mouse models, and robust evidence from human tuberculosis trials is still lacking (Liu et al., 2024; Wang et al., 2024; Luan et al., 2025). Before clinical translation, key issues including long-term safety, biodistribution, reproducible manufacturing, pulmonary delivery efficiency, off-target immune effects and compatibility with standard anti-tuberculosis regimens must be resolved. Thus, future host-directed and nanomedicine-based therapies should be evaluated not only for bacterial killing, but also for their ability to restore balanced macrophage function and preserve protective tissue architecture.

Future studies integrating single-cell transcriptomics, spatial multi-omics and functional validation will be essential to define the molecular and spatial architecture of macrophage states during tuberculosis. Linking these maps to perturbation experiments, bacterial burden and treatment response will be particularly important for distinguishing protective transitional states from permissive tissue-remodeling or foamy macrophage states. Such approaches may ultimately facilitate the development of precision host-directed therapies capable of selectively reshaping pathogenic macrophage programs and improving clinical outcomes in tuberculosis.
